# Supplementary material for: circAMN1-Mediated Ferroptosis Regulates the Expulsion of Placenta in Trophoblast Cells
Source: Antioxidants (Basel). 2024 Apr 11;13(4):451. doi: 10.3390/antiox13040451 (PMC11047571; doi:10.3390/antiox13040451)
Supplement: Supplementary file 1 [file antioxidants-13-00451-s001.zip › Supplementary Table 1.pdf]

Supplementary Table 1. Primer information for circRNAs.

Isoform name	Gene name	Primer sequence-Forward (5′-3′)	Primer sequence-Reverse (5′-3′)
ENSBTAT00000069762	*circRNA1636*	TGAGACCAGTGCTGAGGATG	GGTGACTCCTTTGAGCCAGA
ENSBTAT00000063826	*circRNA1890*	AGCATTATCTGCATACCAGAGG	CAGACTCCACTTTTCCTTCAGC
ENSBTAT00000066850	*circRNA2513*	ATGGCCCCTGAGATGTATGA	CGCTGCCTCTCAGACTTTGT
ENSBTAT00000010553	*circRNA2656*	GATGGTCCATTTCTGGATGG	GCCCCTTGATATCACCTGAA
ENSBTAT00000016319	*circRNA3288*	AGATGGAGCCTTGACACTGG	CAGACTGGGCAACAGCTACA
ENSBTAT00000075440	*circRNA3623*	ATGAAGATTTGGAGCGCAAG	ATCGGAGCTGGAATAAGCAA
intergenic_circRNA	*circRNA3998*	GGCTTCTGCTCAGTTTCACC	AAAGGCAGGCAGTTGTTGAT
intergenic_circRNA	*circRNA4015*	GTATGGAGGGGGTGATTCCT	ATCACTCAGTCCCCACAAGG
ENSBTAT00000035479	*circRNA4517*	CGAGATGTGAAACACCTGGA	GCATTGGTGCTCTGAAGATG
ENSBTAT00000013714	*circRNA4804*	ACCTCCTGCTGACAGGACAC	GTTCCTTCCGCTCTTCCTCT
ENSBTAT00000023577	*circRNA6208*	GCAAGGTCAGCAGGACTTTT	GTTGAAGCAGCACAGTTGGA
ENSBTAT00000072428	*circRNA7697*	CCTGAGCAACCAGAGAAACC	CCGAGTGCTCTGTGTAACCA
ENSBTAT00000018812	*circRNA2480*	TTCATATGGGGCATTGTGTG	GGTGGCAAAGGCTTAATGTC

Isoform name: The linear transcript ID corresponding to circRNA.
