# Supplementary material for: circAMN1-Mediated Ferroptosis Regulates the Expulsion of Placenta in Trophoblast Cells
Source: Antioxidants (Basel). 2024 Apr 11;13(4):451. doi: 10.3390/antiox13040451 (PMC11047571; doi:10.3390/antiox13040451)
Supplement: Supplementary file 1 [file antioxidants-13-00451-s001.zip › Supplementary Table 2.pdf]

Supplementary Table 2. Primer information for mRNAs and miRNAs.

Gene accession number	Gene name	Primer sequence-Forward (5′-3′)	Primer sequence-Reverse (5′-3′)
NM_001034034.2	*GAPDH*	ACCCAGAAGACTGTGGATGG	CAACAGACACGTTGGGAGTG
XM_059887429	*SLC39A8*	TGATCACACCCACTTTGGAA	GGCCCCTTCAAACAAGTACA
XM_059886476.1	*AMN1*	GTCCTTGCAGGCATTAGGAG	GGGCATCCATGGAAGAGTAA
bta-mir-205	*miR-205_R-1*	AGGCGCATTCCTTCATTCCAC	Universal reverse*
NM_001034740	*U6*	CTGTTGCTGTCATTACTTCTGATG	Universal reverse*

* Universal reverse was provided by the manufacturer (Evo M-MLV RT Kit with gDNA Clean for qPCR, Accurate Biology, Changsha, Hunan, China).

bta-mir-205: The registration number of the mature miRNA sequence of the species on the matching miRBase database.
