# Supplementary material for: circAMN1-Mediated Ferroptosis Regulates the Expulsion of Placenta in Trophoblast Cells
Source: Antioxidants (Basel). 2024 Apr 11;13(4):451. doi: 10.3390/antiox13040451 (PMC11047571; doi:10.3390/antiox13040451)
Supplement: Supplementary file 1 [file antioxidants-13-00451-s001.zip › Supplementary Table 3.pdf]

Supplementary Table 3. circRNA and miRNA RNA sequences.

Gene ID	Gene name	Sense (5′-3′)	Antisense (5'-3')
ENSBTAT00000018812	circAMN1 1#	UAACAGAUGCCUUUGGUGCTT	GCACCAAAGGCAUCUGUUATT
	circAMN1 2#	CUCUAAUAACAGAUGCCUUTT	AAGGCAUCUGUUAUUAGAGT T
bta-mir-205	miR-205_R-1 mimic	UCCUUCAUUCCACCGGAGUCU	ACUCCGGUGGAAUGAAGGAU U
	mimic NC	UUGUACUACACAAAAGUACUG	GUACUUUUGUGUAGUACAAU U
	bta-miR-205_R-1 inhibitor	(mA)(mG)(mA)(mC)(mU)(mC)(mC)(mG)(mG)(mU)(mG)(mG)(mA)(mA)(mU)(mG)(mA)(mA)(mG)(mG)(mA)	/
	inhibitor NC	CAG UAC UUU UGU GUA GUA CAA	/

ENSBTAT00000018812: The linear transcript ID corresponding to circRNA.

bta-mir-205: The registration number of the mature miRNA sequence of the species on the matching miRBase database.
